# Beta Oscillations and Pupil-Linked Arousal Regulate the Updating of Cognitive Models

**DOI:** 10.1523/JNEUROSCI.0415-26.2026

**Published:** 2026-08-24

**Authors:** Charlotte R. Marshall, Mengxi Wang, Emma L. Lawrance, Nils Kolling, Jill X. O’Reilly

**Affiliations:** ^1^Department of Experimental Psychology, University of Oxford, Oxford OX1 3EL, United Kingdom; ^2^Centre for Human Brain Health, School of Psychology, University of Birmingham, Birmingham B15 2TT, United Kingdom; ^3^Faculty of Medicine, Department of Surgery & Cancer, Imperial College London, London SW7 2AZ, United Kingdom; ^4^Institute of Global Health Innovation and Grantham Institute for Climate Change and the Environment, Imperial College London, London SW7 2AZ, United Kingdom; ^5^Climate Cares, Department of Psychiatry, University of Oxford, Oxford OX37JX, United Kingdom; ^6^Université Lyon 1, INSERM, Stem Cell and Brain Research Institute, Lyon 69500, France; ^7^Centre Hospitalier Le Vinatier, Pôle EST, Bron 69678, France

**Keywords:** Bayesian update, beta oscillations, pupillometry, uncertainty

## Abstract

The beta rhythm (neural oscillations in the range 13–30 Hz) is associated with the control of movement. Discrete movements (such as a button press) are bounded by a premovement dip in beta power and a postmovement beta rebound, which are thought to release and terminate the action, respectively. However, an additional or alternative role has been suggested for the beta rebound in regulating the extent to which sensory feedback updates the task model. Using EEG and pupillometry in human volunteers of both sexes, we developed a task that dissociates updating of a cognitive model from motor control. We found that beta oscillations were modulated in two independent modes—phasic and tonic—with distinct computational correlates. When abstract task models are updated by integrating the outcomes of several actions, there is a tonic suppression of the beta rhythm, which originates at the time of the postmovement rebound, spans multiple trials, and is measurable at both the pre- and postmovement stages. Independently, phasic trial-to-trial fluctuations in beta power, primarily measurable in the premovement phase, were associated with motor readiness. The decrease in tonic beta power during model updating was mediated by an increase in tonic pupil dilation, a known correlate of model updating often considered a proxy for neuromodulatory tone.

## Significance Statement

Beta oscillations have traditionally been linked to movement but are increasingly implicated in learning and cognitive control. We show that these functions are supported by two computationally distinct modes of beta activity. A transient, phasic component reflects motor readiness immediately surrounding voluntary actions. In contrast, a sustained, tonic suppression of beta develops over multiple trials as evidence accumulates that an internal model of the environment should be updated. This tonic signal emerges from the postmovement beta rebound, tracks Bayesian estimates of state uncertainty, and parallels tonic pupil-linked arousal. These findings unify motor and cognitive accounts of beta oscillations by demonstrating that beta supports distinct computations on separate timescales.

## Introduction

Sensorimotor beta oscillations (13–30 Hz) arise over the frontal cortex and are reliably observed during voluntary movements (Jasper and Penfield, 1949). While classic models suggest that beta oscillations gate the release of movement, more recent research suggests a role, specifically for postmovement beta, in controlling the learning rate for higher-level sensorimotor models (the degree to which sensory feedback updates models), and theory suggests a more extensive role in controlling the update of cognitive models (Engel and Fries, 2010).

During punctate, voluntary movements such as button presses, beta power follows a characteristic temporal motif: a premovement dip (Pfurtscheller, 1981), followed by a postmovement rebound (Pfurtscheller et al., 1996), both showing high intraindividual reliability (Espenhahn et al., 2017). Beta oscillations are inhibitory of movement, for example, in Parkinson's disease (Kuhn et al., 2004). Therefore, an interpretation of the beta motif is that premovement beta dip “releases” actions (by suppressing the holding beta rhythm), while a postmovement beta rebound terminates each action.

More recently, evidence suggests that beta oscillations not only gate the release of movement but control the rate of updating of high-level motor plans by feedback (Gilbertson et al., 2005), that is, the local learning rate. In particular, the postmovement beta rebound is well timed to block sensory feedback, resulting from one's actions, from updating the sensorimotor model for future occasions. In motor learning, the beta rebound is reduced in conditions requiring update of a behavioral model, such as following large-magnitude movement errors in a visuomotor adaptation task (Tan et al., 2014), especially when confidence in the world model is low because the environment is unpredictable (Tan et al., 2016) and when sensory feedback is more informative because it is less noisy (Palmer et al., 2019). Similarly, occipital beta power decreases when visual feedback is to be used and increases when it is to be ignored (Limanowski et al., 2020). Taken together, these results suggest that the phasic beta rebound acts at the time of feedback to block (or permit) the updating of a motor plan or model, thus acting at the level of motor planning rather than the immediate control of movement.

Despite this evidence, two important questions remain. First, although Engel and Fries (2010) proposed a general role for beta oscillations in maintaining or updating cognitive models, most existing work has focused on sensorimotor adaptation. Second, it remains unclear whether update-related beta changes are necessarily confined to the phasic postmovement rebound. In many cognitive tasks, model updating depends on integrating evidence across multiple trials rather than responding to a single feedback event (Wilson et al., 2013). If learning rate remains elevated across successive trials, beta activity might also show sustained, tonic changes. Indeed, Engel and Fries proposed that tonic beta activity promotes internally driven processing, whereas reduced tonic beta favors stimulus-driven updating.

Here, we used a task that dissociates updating beliefs about the external environment from motor execution and perceptual errors. This allowed us to distinguish the multiple factors associated with the beta rhythm, across multiple timescales: motor readiness, feedback processing relating to the current action and sensory stimulus, and higher-level uncertainty about a model of the state of the world. This is distinct from previous research which has generally used visuomotor tasks, in which model feedback, model updating, and uncertainty tend to be highly correlated. We used a hierarchical perceptual decision-making task, in which uncertainty varied independently at the level of individual decisions and at the (higher) level of the participant's beliefs about the current state of the world. We recorded the beta rhythm using whole-head EEG and report distinct computational effects on the beta rhythm in the phasic and tonic timescales. These effects were mirrored in parallel modulation of pupil dilation on the phasic and tonic timescales, linking tonic suppression of beta oscillations to an existing literature in which pupil-linked arousal increases around change points in the environment.

## Materials and Methods

### Participants

Thirty-six healthy right-handed participants (aged 18–40 years), both male and female, took part in the study, which was approved by the University Research Ethics Committee. Participants were recruited from a student population at the University of Oxford, and all gave written consent using a form approved by the ethics committee. All participants completed the total 360 trials of the experiment. Nine participants were excluded from analyses due to frequent eye movements (in over 25% of trials, their gaze deviated by more than 200 pixels on the *x*-axis from the screen center), resulting in a final sample size of 27. Participants received reimbursement for their time upon completion of the experiment.

### Experimental design and statistical analysis

#### Task

To dissociate between immediate feedback on a proximal action and higher-level model updating, we designed a hierarchical perceptual decision-making task in which negative feedback on a given trial could be attributed either to (one's own) poor performance or to a mismatch between the expected and actual generative state of the environment.

Participants performed a hierarchical orientation judgment task (Fig. 1*a*). On each trial, participants made a perceptual decision: they judged the direction of rotation between two (sequential) grating stimuli; the difficulty of this judgment (angle of rotation) varied from trial to trial (Fig. 1*b*).

**Figure 1.**
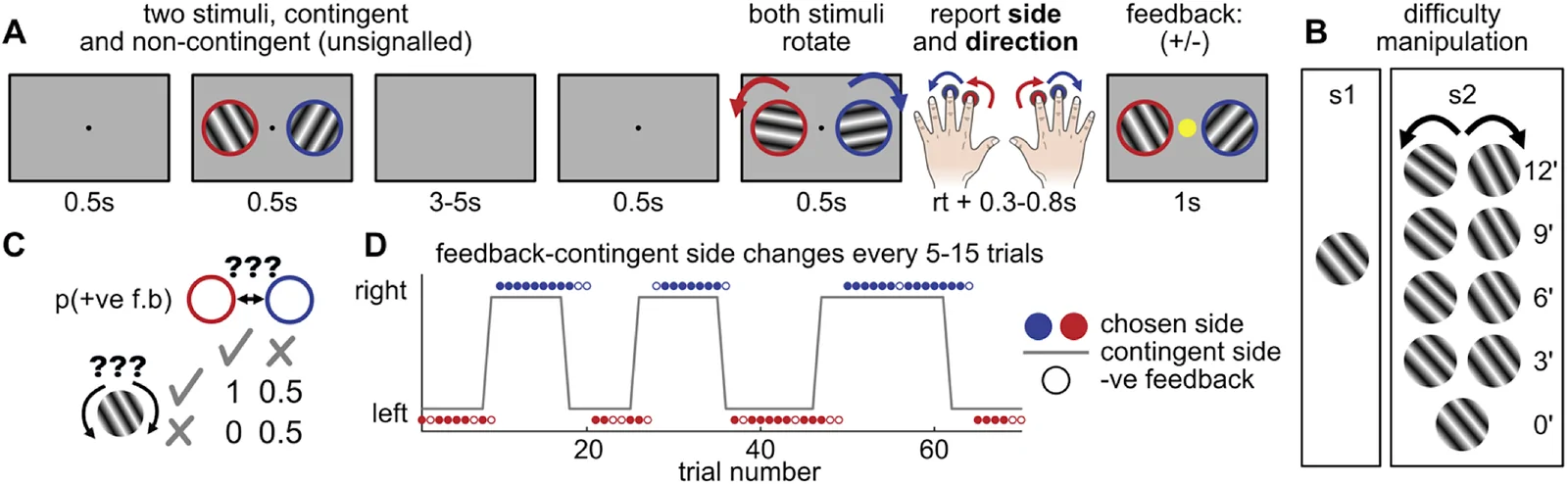
Behavioral task. ***a***, Structure of a single trial; the mean total trial duration is 12 s, including an ITI of 4 s. ***b***, Example difficulty manipulation: Second stimuli (s2) could rotate either clockwise or counterclockwise relative to first stimuli (s1). Rotations could range from 3 to 12° or remain unchanged (0° rotation). ***c***, Outcome probabilities on a given trial. Of the four possible choices, only one guaranteed positive feedback, selecting both the correct side and the correct direction of rotation. Perceptual errors resulted in negative feedback. On trials with zero rotation, one answer was arbitrarily assigned as correct, and participants were given feedback accordingly. Side errors resulted in positive or negative feedback, at chance level. ***d***, Example sequence of reward-contingent sides (solid gray line) and side chosen by participant (red and blue dots). Feedback is indicated by dot fill; unfilled dots indicate negative feedback at the end of that trial. The magnitude (but not direction) of the rotation was matched for left- and right-hand stimuli on each trial to preclude a strategy in which participants monitored both stimulus pairs and responded to the one with the easier perceptual decision.

*State inference task.* The interpretation of feedback on each trial was complicated by a further factor: out of a choice of two stimulus pairs (left side, right side), only one gave performance-contingent feedback (Fig. 1*c*). On each trial, participants chose to respond to the left or right stimulus pair. If the selected pair was the contingent pair, correct responses were always met with positive feedback. If the selected pair was the noncontingent pair, positive or negative feedback was given at random. Which side (left/right) was contingent was stable for 5–15 trials at a time and then reversed without warning (state reversal). We refer to the task of inferring which stimulus pair was contingent as the state inference task. Note that participants were instructed about this manipulation before the task and given explicit practice with feedback to ensure they understood that only one stimulus gave contingent feedback at a given time.

*Interpreting feedback*. Because there was uncertainty at the perceptual level and the state level, when negative feedback was received, participants needed to decide whether this indicated a perceptual error (incorrect rotation reporting) or that they were working on the noncontingent stimuli, in other words, that their model of the environment was inconsistent with its current state. A single instance of negative feedback was generally insufficient for a participant to conclude that the contingent side had changed, since negative feedback could also be attributed to a perceptual error. Therefore, maximizing reward necessitated tracking feedback history over several trials, building up evidence for a decision to switch sides in the case of repeated negative feedback (“behavioral state reversal”; Fig. 1*d*). This manipulation allowed us to investigate changes in beta power during extended periods of state uncertainty or updating, as distinct from individual feedback events.

#### Task details: trial structure

Each trial began with the presentation of a central fixation dot for 0.5 s, followed by two Gabor patches oriented randomly between 30 and 60° to the left and right of fixation on the horizontal midline subtending 18.3° of visual angle for 0.5 s. After a variable working memory interval (also called interstimulus interval or ISI) of 3–5 s, mean 4 s, a second fixation dot (0.5 s) preceded re-presentation of the two gratings (0.5 s), both rotated either clockwise or counterclockwise from their initial positions by 0° (i.e., unchanged), 3, 6, 9, or 12°. The magnitude of rotation was the same for both gratings, but the direction of rotation was random. Participants chose a response from four buttons, indicating both the direction of rotation and the stimulus (left or right) to which they were responding; this allowed us to monitor their belief about the contingent side as well as perceptual accuracy. There was a variable jitter of 300–800 ms; then feedback was indicated by a color change at the central fixation dot (1 s). There was an ITI of 4 s giving an average total trial duration of 12 s.

Each participant completed three runs of the orientation judgment task, consisting of 120 trials, for a total of 360 trials.

### Behavioral data analysis

#### Single-trial predictors of perceptual accuracy and reaction time

We expected that participants would be more likely to give a “perceptually correct” answer (one in which the direction of rotation was reported correctly) and would show faster reaction times, when the angle of rotation between the first and second stimulus was larger and the working memory delay was shorter. We used multiple logistic and linear regression, respectively, with two explanatory variables (angle of rotation α and duration of working memory interval, ISI) to test these hypotheses:

p(choose"clockwise")∼[constant+α+signedISI].(1)

(Logistic regression for probability of perceptually correct response)

Note that the response variable here was whether the participant indicated clockwise rotation regardless of whether the feedback-contingent side was correctly chosen, which we have termed a “perceptually correct” response. The explanatory variables were angle of rotation (here and only here, signed as positive for clockwise rotations) and the working memory interval or ISI, here and only here modified by a sign so that the prediction is positive (more likely to respond “clockwise”) for long intervals when the correct response is clockwise, and the converse (more likely to respond “counterclockwise”) for long intervals, when the correct response is counterclockwise. This is to allow a logistic curve to be fit to the binary response clockwise/counterclockwise.

For the analysis of reaction times, we used a linear regression:

RT∼[constant+α+ISI].(2)

(Linear regression predicting reaction time based on task factors)

In this case, the unsigned angle of rotation (i.e., difficulty) and working memory interval were used as predictors.

#### Single- and cross-trial predictors of switching

We expected that participants would (as instructed) attempt to choose the feedback-contingent side on each trial but that there would be some delay after the contingent side reversed before participants switched responding to the new contingent side due to the need to accumulate evidence.

We modeled the probability of a behavioral state reversal (i.e., the participant switching which side they respond to) after feedback on trial *t* using a lagged logistic regression with binary explanatory variables that captured the presence of negative feedback on previous trials as follows:

EV1: $x_{t}^{\prime }=1\;\mathrm{if}\;(x_{t}=0),$
EV2: $x_{t-1}^{\prime }=1\;\mathrm{if}\;(x_{t}=0\;\mathrm{and}\;x_{t-1}=0),$
EV3: $x_{t-2}^{\prime }=1\;\mathrm{if}\;(x_{t}=0\;\;\mathrm{and}\;x_{t-1}=0\;\mathrm{and}\;x_{t-2}=0),$
EV4: $\;x_{t-3}^{\prime }=1\;\mathrm{if}\;(x_{t}=0\;\mathrm{and}\;x_{t-1}=0\;\mathrm{and}\;x_{t-2}=0\;\mathrm{and}\;x_{t-3}=0).$ (3)

(Explanatory variables for lagged regression predicting side switch)

The sequence was concluded if there was a behavioral state reversal (i.e., the participant switched which side they selected) at any point. In addition, we included EVs representing the perceptual difficulty (denoted as α, the angle of rotation between Stimuli 1 and 2) on each negative-feedback trial, as we expected that negative feedback would have a greater influence on state reversals when the perceptual judgment was easy (and the observer should have high confidence in their prediction). Difficulty was only modeled for trials with negative feedback, as follows:

$$\alpha_{t}^{\prime }=\{\begin{array}{cc} \alpha_{t} & \mathrm{if}\;{x^{\prime }}_{t}=1\\ 0 & \mathrm{otherwise} \end{array}.\;(4)$$

(Parametric modulation by difficulty)

Thus the complete regression model was as follows:

$$\begin{array}{rl} p(\mathrm{switch})\;∼\;[ & \mathrm{constant}+\;{x^{\prime }}_{t-1}+\;{x^{\prime }}_{t-2}+{x^{\prime }}_{t-3}\\ & +\;\alpha_{t}+\;\alpha_{t-1}+\;\alpha_{t-2}+\alpha_{t-3}]. \end{array}\;(5)$$

(Logistic lagged regression predicting side switch)

*Bayesian model*. To illustrate how an ideal observer would accumulate evidence for a state reversal, we modeled the evidence in favor of each side being the deterministic side using a Bayesian learner model. The model gives the “evidence for/against side,” which will be plotted in Figures 2*d* and 3*d*.

**Figure 2.**
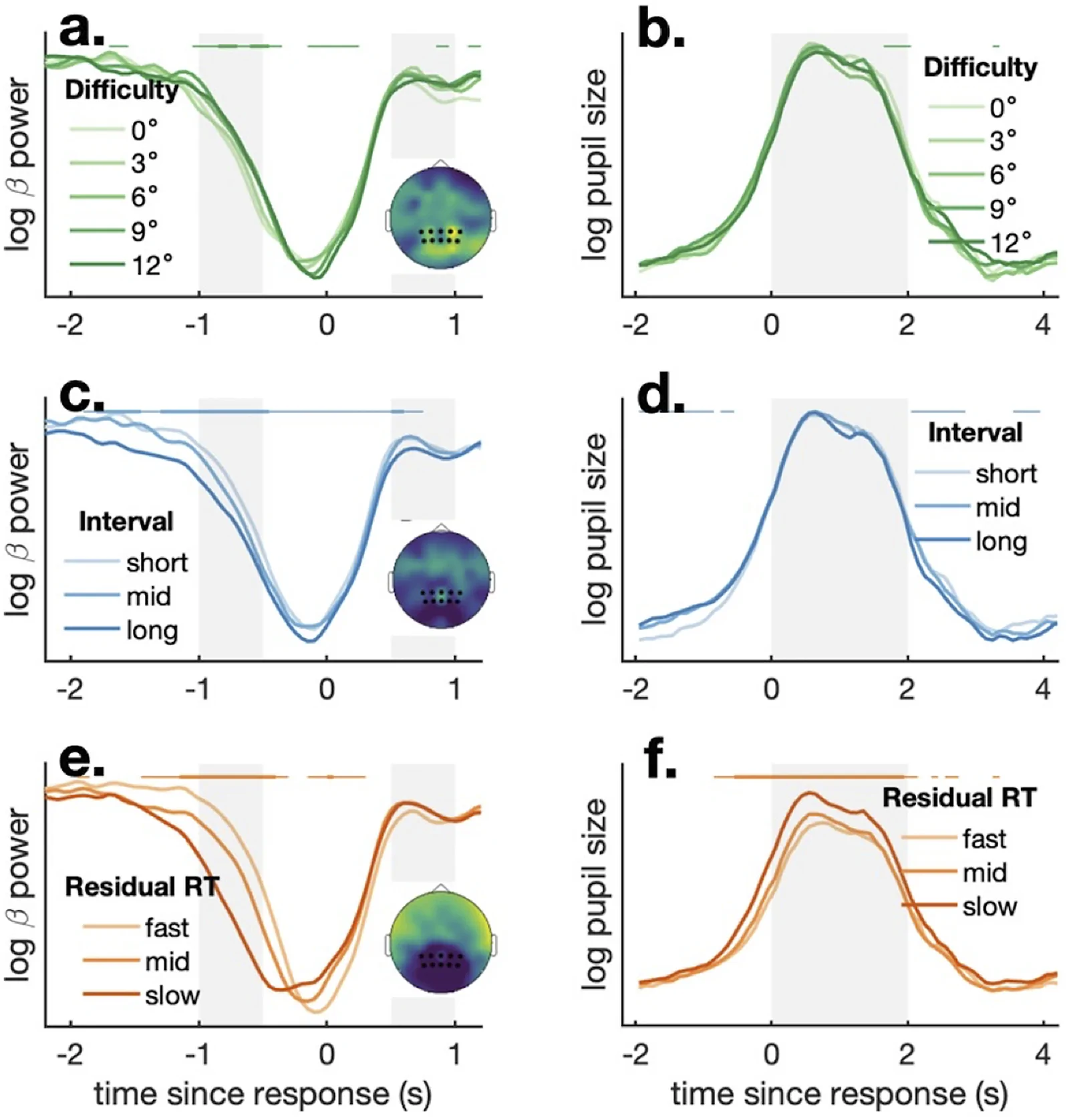
Response readiness is reflected in decreased phasic pupil response and premovement beta power but has no effect on postmovement beta power or tonic pupil dilation. ***a, c, e***, Beta-band power, time-locked to the button-press response. Premovement beta power was lower when (***a***) difficulty was low, (***c***) the working memory interval was longer, and (***e***) the reaction time was faster than expected given difficulty and working memory interval. ***b, d, f***, Pupil dilation, time-locked to the button-press response. Units are arbitrary. The phasic pupil response to the button press (a transient dilation lasting ∼2 s after an event) is larger on trials with slower reaction times than expected, given difficulty and working memory interval. There was no effect of task difficulty or ISI on pupil size. In panels ***c–f***, tertile splits are shown for illustration only; the statistical analysis was a time-resolved GLM using continuous measures. In all panels, horizontal lines indicate time points at which the effect of each explanatory was statistically significant *t* test of *β* values from individual participant GLMs, at each time point against zero [26 df; *α* = 0.05, two tailed; Bonferroni-corrected for 81 timepoints (thick line) or uncorrected (thin line)].

**Figure 3.**
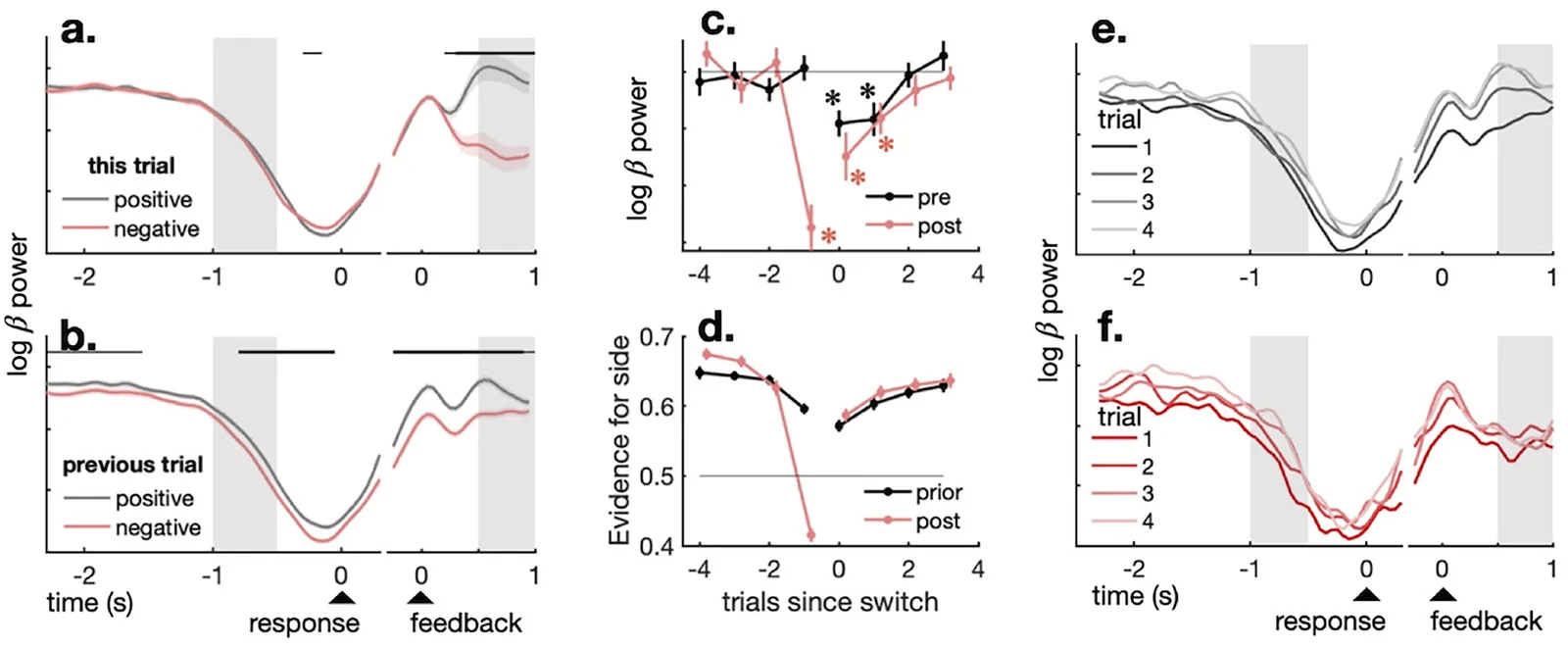
Beta power is tonically reduced around the point of state transition. ***a***, Following negative feedback (red), beta power immediately decreases. ***b***, Beta power remains tonically lowered throughout the trial following negative feedback. **Horizontal lines** indicate the periods in which there is a statistically significant reduction in beta power associated with negative feedback on current trial (top) or previous trial (bottom); *p* < 0.05 one-tailed uncorrected (fine lines) and Bonferroni-corrected for 81 timepoints (heavy lines). ***c***, Around a state reversal (side switch), the phasic beta response to feedback **(red markers)** is affected; there is a large decrease in postfeedback beta on the final trial before the side switch, and beta power in the postfeedback window returns to baseline gradually over several trials following the side switch. The reduction in beta power following the side switch is sustained across the whole trial; the presented values **(black markers)** are the average beta power in the time window 1–0.5 s before the movement on each trial. The windows used for the red and black trace are shaded in parts ***a***, ***b***, ***e***, and ***f*** .Time points significantly different to the overall mean are marked with a star (*p* < 0.05 corrected for 8 comparisons). ***d***, The tonic reduction in beta power shown in part c mirrors the strength of evidence favoring a side switch, obtained from a Bayesian ideal observer model (see Materials and Methods)—*y*-axis shows the probability that the chosen side is indeed the correct (deterministic) side on each trial. Note that the posterior probability indeed falls below 0.5 (i.e., favors the nonchosen side), only on the trial directly preceding the switch—however, the belief that one is choosing the correct side starts to fall ahead of the switch and rises slowly over several trials after the switch. ***e, f***, Illustrative plot showing the full beta motif on the four trials after a side switch [divided into trials with positive (***e***) and negative (***f***) feedback]. Note the tonic reduction in beta is evident across the whole time window.

The hypothesis space of the model was a simple binary: [L,R], representing the belief that the left or right stimulus pair is feedback contingent, respectively. Because exactly one stimulus pair was feedback contingent on each trial, the participant's prior belief $p(L_{t}|x_{0:t-1})$ and posterior belief $p(L_{t}|x_{0:t})$ were scalars.

On a given trial t, the likelihood $p(L_{t}|x_{t})$ was equivalent to the probability of obtaining the given feedback, given the angle of rotation and working memory delay on that trial and assuming that L was in fact true. This was obtained for each individual using logistic regression (as described above) as follows:

$$p(L_{t}|x_{t})=\;p(x_{t}|L_{t})\;∼\;(\alpha +\mathrm{ISI}),\;(6)$$

(Likelihood function of a Bayesian model)

where *α* is the angle of rotation between first and second stimuli, determining task difficulty, and ISI is the duration of the working memory interval.

The posterior belief $p(L_{t}|x_{0:t})$ was then obtained using Bayes' rule:

$$p(L_{t}|x_{0:t})\propto \;p(L_{t}|x_{t})\cdot p(L_{t}|x_{0:t-1}).\;(7)$$

(Bayesian update)

The prior on the following trial was obtained from the posterior, using a simple leak that reflected the true frequency of reversals, H=(1/10):

$$p(L_{t+1}|x_{0:t})=(\;p(L_{t}|x_{0:t})\cdot (1-H))+((1-p(L_{t}|x_{0:t}))\cdot H).\;(8)$$

(Transition function from a Bayesian model)

The prior on the first trial of the run was set to 0.5.

The “evidence for side” plotted in Figure 3*d* is the probability that the chosen side is indeed the correct side (noting that the chosen side changes at the point of the switch). The “evidence against side” in Figure 4*d* is the complement of “evidence for side”—shown this way to match the polarity of the pupil effect and therefore aid visual comparison.

**Figure 4.**
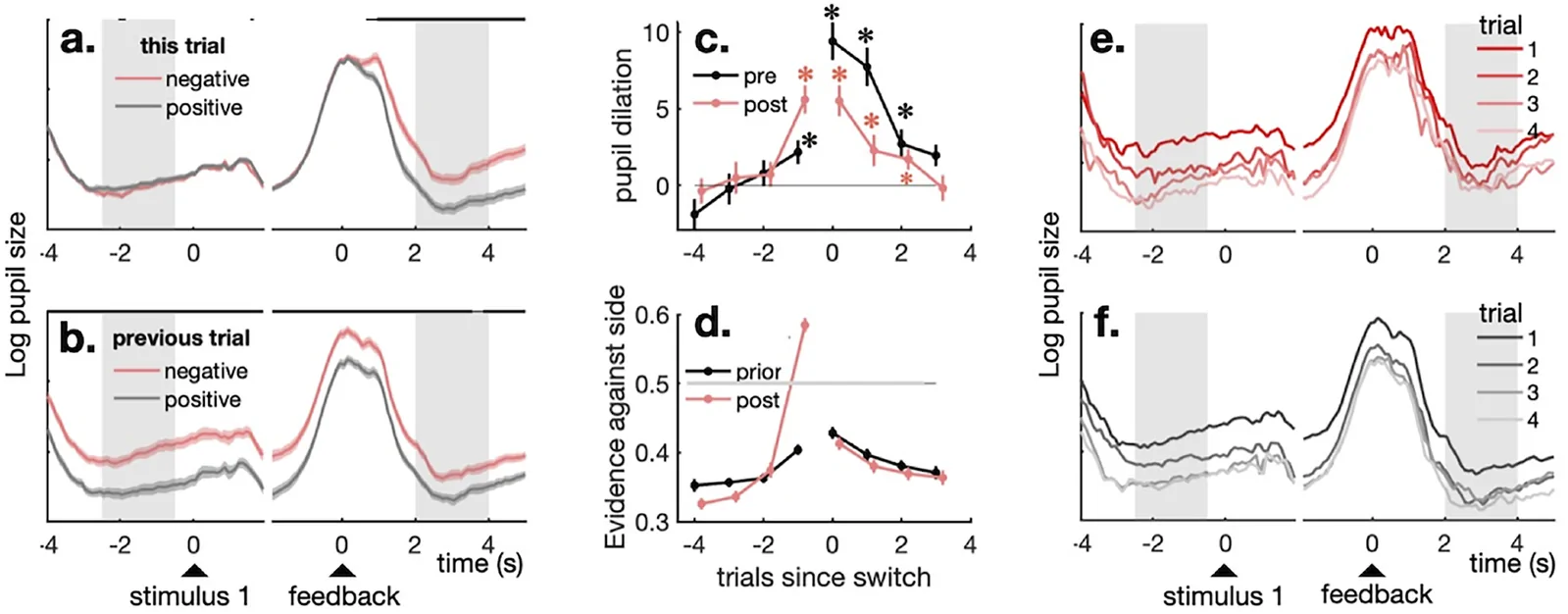
Pupil size is tonically increased around the point of state transition. ***a***, Following negative feedback (red), pupil size immediately increases. ***b***, Pupil size remains tonically raised throughout the trial following negative feedback. **Horizontal lines** indicate the periods in which there is a statistically significant increase in pupil size associated with negative feedback on current trial (top) or previous trial (bottom); *p* < 0.05 one-tailed uncorrected (fine lines) and Bonferroni-corrected for 81 timepoints (heavy lines). ***c***, Around a state reversal (side switch), the phasic pupil response to feedback **(red markers)** is affected; there is a large increase in postfeedback pupil size on the final trial before the side switch, and pupil size in the postfeedback window returns to baseline gradually over several trials following the side switch. The increase in pupil size following the side switch is sustained across the whole trial; the presented values **(black markers)** are the average pupil size in the time window 1–0.5 s before the movement on each trial. The windows used for the red and black trace are shaded in parts ***a***, ***b***, ***e***, and *** f***. Time points significantly different to the overall mean are marked with star (large star, *p* < 0.05 corrected for 8 comparisons; small star *p* < 0.05 uncorrected). ***d***, The tonic increase in pupil size shown in part c mirrors the strength of evidence favoring a side switch, obtained from a Bayesian ideal observer model (see Materials and Methods)—*y*-axis shows the probability that the nonchosen side is indeed the correct (deterministic) side on each trial. Note that the posterior probability indeed rises above 0.5 (i.e., favors the nonchosen side), only the trial directly preceding the switch—however, the belief that one should choose the nonchosen side starts to rise ahead of the switch and falls slowly over several trials after the switch. Note that the values plotted here are simply the complement of Figures 3*d* and 7*d*, shown in this orientation to facilitate comparison with the pupil size effect. ***e, f***, Illustrative plot showing the full pupil dilation motif on the four trials after a side switch [divided into trials with positive (***e***) and negative (***f***) feedback]. Note the tonic increase in pupil size is evident across the whole time window.

#### EEG acquisition

During the orientation judgment task, EEG was recorded using a 61-channel 10–20 Easycap active EEG cap (Easycap) and SynAmps RT Amplifier (Compumedics), at 1 kHz, online referenced to the right mastoid.

#### EEG analysis

All EEG analyses were conducted in FieldTrip (Oostenveld et al., 2011). Data were imported, band-passed at 1–40 Hz, and re-referenced to linked mastoids.

Independent component analysis (ICA) cleaning was first performed on continuous data. For every participant, data from the three EEG runs were divided into 1 s epochs, concatenated, and then manual inspection was used to identify “bad” EEG segments (function ft_rejectvisual.m, manual rejection based on epoch variance). ICA (method “runica”; Bell and Sejnowski, 1995) was then performed on all data epochs not marked as “bad.” Data were downsampled to 250 Hz, and PCA was used to reduce the rank of the data to 40 to speed up ICA. The first 20 ICA component time courses were then correlated with the horizontal and vertical EOG time courses to identify components containing primarily ocular artifacts. Components with an overall correlation of above 0.25 were marked for removal. This led to between one and four ICA components being marked for rejection per participant (mean, 2.2).

The original raw data were then re-epoched into 5 s trials around the button-press events (from 2.5 s prepress to 2.5 s postpress). These trial-based data were downsampled to 500 Hz, and the ICA unmixing weights from the continuous data were used to remove identified ICA components. Scalp current density [surface Laplacian (Oostendorp and van Oosterom, 1996)] was estimated to increase spatial specificity of the EEG data. Time–frequency analysis was then performed for integer frequencies from 1 to 40 Hz using a 400 ms sliding window multiplied with a Hanning taper moving across the entire 5 s epoch in 50 ms increments.

We then averaged over a broad beta band of 16–24 Hz over sensors CP3, CP1, CPz, CP2, CP4, P3, P1, Pz, P2, and P4. Although beta power is sometimes defined as 13–30 Hz, we focused on the slightly narrower 16–24 Hz band to minimize contamination from adjacent bands (in particular the alpha band; Fig. 5) and because inspection of the all-trial grand average suggested that this range contained the bulk of perimovement beta modulation.

**Figure 5.**
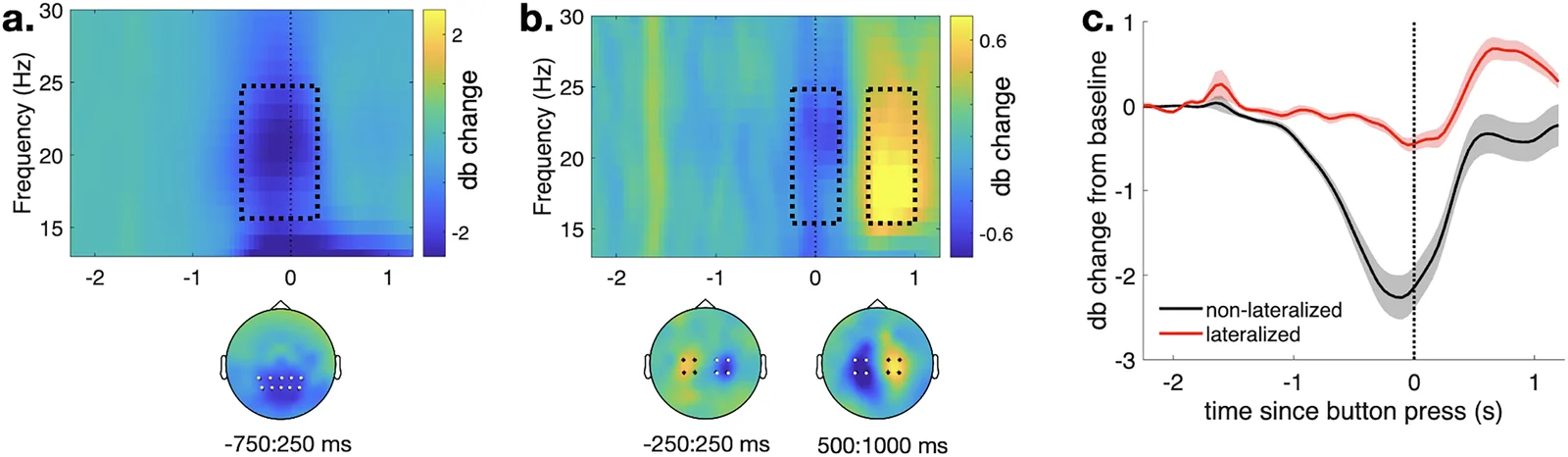
Lateralized and nonlateralized beta power grand averages. ***a***, Average beta-band power averaged over all trials, without correction for hand of response. Box shows the time window used for the topo plot. Time windows were selected based on the time–frequency diagram and are therefore illustrative only. ***b***, Average lateralized beta-band power corrected for hand of response, at ipsi- and contralateral central sensors (C1/2, C3/4, and CP1/2, CP3/4). Boxes show the time–frequency windows used for the topo plots; time windows were selected based on the time–frequency diagram and are therefore illustrative only. Topo plots: topographies on right-hand response trials are left–right reversed to allow for averaging. ***c***, Time course of beta-band (16–24 Hz) activity, nonlateralized as in ***a*** and lateralized as in ***b***. Qualitatively, the nonlateralized beta dip appears to precede the lateralized effect. For this visualization, power was calculated as decibel change from baseline at −2,250:−1,750 ms before the response.

While beta power is often studied as a lateralized phenomenon, in the current task, by design, the response hand could not be predicted on a given trial in advance of the actual response. Therefore, we did not expect to see lateralized effects of state inference. For completeness, we show here topographies for lateralized and nonlateralized beta-band power. It can be seen that when all trials are averaged, the peak perimovement beta dip occurs in the range 16–24 Hz at the CP and P sensors. In contrast, when beta power is plotted as a difference between sensors contra- and ipsilateral to the hand of response, the beta motif is seen at more anterior sensors (the C and CP sensors) and slightly later in time. The analysis presented hereafter focuses on nonlateralized beta power—to preview the results, no task variables of interest were found to significantly predict lateralized beta power.

#### Pupil data acquisition

Pupil dilation was tracked in a single eye at 1,000 Hz, using an EyeLink 1000 optical eye tracker (SR Research). Pupil data were preprocessed using custom software implemented in MATLAB. Data were smoothed with a Gaussian kernel (FWHM 50 ms). Blinks were detected as rapid downward deflections in the pupil size trace; blinks plus a 75 ms buffer either side were coded as missing data and excluded from further analysis.

## Results

We first present behavioral analysis confirming that our manipulation of perceptual difficulty was effective and that participants integrated feedback history with perceptual difficulty when detecting state reversals, before moving on to examine neural signals.

### Both accuracy and reaction time depend on perceptual difficulty

We first confirmed that the perceptual difficulty manipulation was effective. Decreasing the magnitude of the angle between the first and second stimuli produced significant performance deficits; it both decreased the probability of correct response (Fig. 5*a*) and increased mean reaction time (Fig. 5*b*). In a logistic regression (see Eq. 1 in Materials and Methods) the effect of the signed angle of rotation on the reported direction of rotation was significant: across individuals, the mean effect was large: a 27% increase in the odds ratio for choosing the correct side, for each additional degree of rotation. This was statistically significant (sign-rank test for *β* values across the group; *p* = 5.6 × 10^−6^). We included the duration of the working memory interval (ISI) as a covariate as we reasoned longer intervals could lead to lower accuracy due to forgetting; however, the effect of ISI was near zero (0.6% change in the odds ratio for each additional second of ISI) and not statistically significant (*p* = 0.67, sign-rank test). Both angle magnitude and ISI significantly predicted reaction time (linear regression; see Eq. 2 in Materials and Methods): mean effect size across individuals for angle magnitude is −7.5 (7.5 ms decrease in reaction time for each additional degree of rotation), which was statistically significant. For ISI the mean effect size was −8.9 × 10^−3^ that is just under 1 ms decrease in reaction time for each 1 s increase in ISI; although small, representing <5 ms difference in RT between the longest and shortest ISIs, this effect was statistically significant (*t*_(26)_ = −2.30; *p* = 0.029). The effects of decreasing angle magnitude on accuracy and reaction time were correlated across participants (*r*_(26)_ = −0.79; *p* = 7.9 × 10^−7^). No such correlation was observed for the effects of ISI on accuracy and reaction time (*r*_(26)_ = 0.028; *p* = 0.89). Since the above implies that the difficulty manipulation was effective, we expected participants to behave differently on easy versus difficult trials, when weighing up decisions to switch sides.

### Participants integrated feedback history, but not perceptual difficulty history, into decisions to switch

Participants were significantly more likely to switch following a run of negative feedback and were more likely to switch following negative feedback on an easy than a difficult trial, indicating that participants weighed both factors when evaluating whether to switch sides.

Statistically, we tested for these effects using a lagged logistic regression (see Eqs. 3–5 in Materials and Methods), predicting the probability of a side switch based on negative feedback on the current and previous three trials, and the interaction of feedback with angle of rotation (inverse difficulty; i.e., the angle of rotation was modeled for negative-feedback trials only). There was a significant effect of negative feedback on the current and previous trials. Median odds ratios for significant effects were as follows: participants (*n* = 27) were 56.1 times more likely to switch following negative than positive feedback on the current trial (*p* value from the sign-rank test on group *β* values = 5.6 × 10^−6^); indeed 95.4% of switches were on negative-feedback trials. Furthermore, participants were on average 2.26 times more likely to switch if there was, additionally, negative feedback on the previous trial (*p* value from sign-rank test on group *β* values = 4.42 × 10^−5^). The average number of trials completed between a ground-truth reversal in the relevant side and a behavioral switch is 7.4 (SD of participants means, 1.54) or 7.7 (SD, 1.72), if limiting the analysis to “correct” switches in which the participant really switches from the relevant to irrelevant side.

The greater the rotation between stimuli (on the current trial), the more likely participants were to switch (Fig. 6*c*)—they were 1.11 times more likely to switch for each additional degree of rotation (*p* value from sign-rank test on group *β* values = 1.37 × 10^−5^). The effect of negative feedback on trials *t*−2 and earlier and of angle of rotation on all previous trials (*t*−1 and earlier) was nonsignificant (*p* > 0.1).

**Figure 6.**
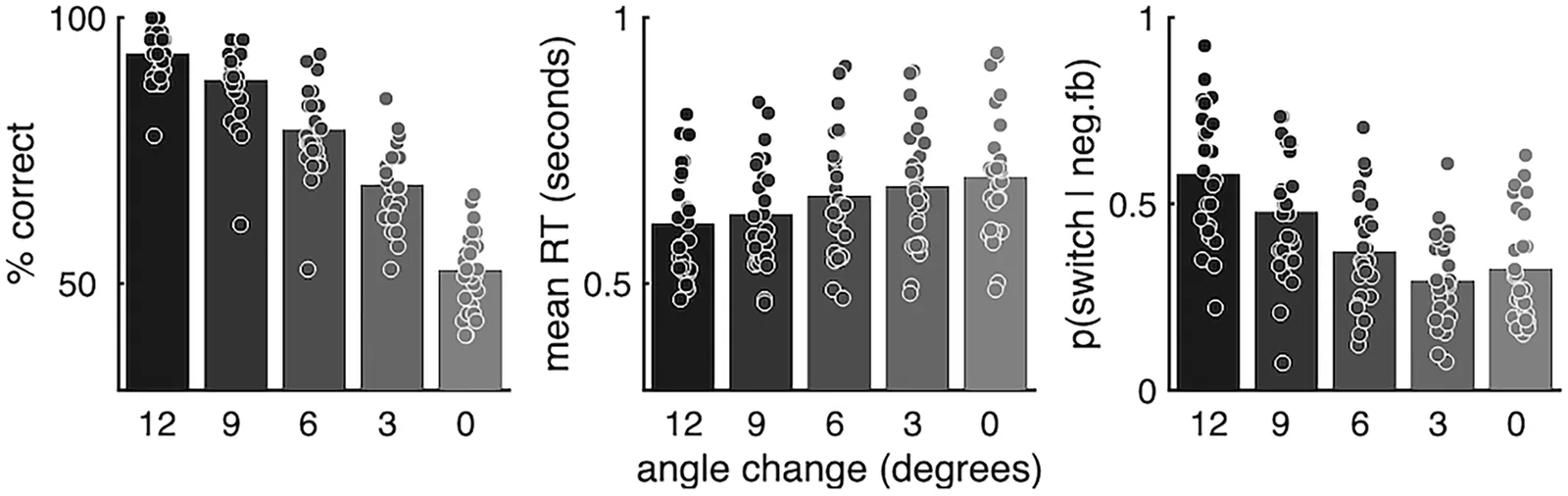
Effects of perceptual difficulty on behavior. ***a***, Change in the percentage of perceptually correct responses as a function of angle difference between Stimuli 1 and 2. Dots represent individual participants. Note that larger angle changes equate to easier trials, i.e., the most difficult trials are plotted on the right side. ***b***, As ***a*** but change in mean reaction time. Dots represent individual datapoints. ***c***, Given that a trial *t* was unrewarded, the probability of switching decreases with increasing perceptual difficulty on trial *t*.

This interplay between switching, feedback, and perceptual difficulty is logical; it suggests participants viewed the negative feedback on a difficult trial as ambiguous (possibly due to either a perceptual error or a side error) but were more likely to attribute negative feedback on an easy trial to a side error. The results suggest that, while feedback on the current trial is weighted by difficulty, feedback from previous trials may be maintained simply as binary outcomes (positive or negative)—although it should be noted that the lack of a historical effect of trial difficulty cannot be regarded as conclusive, as it could reflect a lack of sensitivity.

### Reaction times decrease before a switch but accuracy is maintained

Figure 7 shows reaction times, accuracy, and frequency of positive feedback around the time of a switch. Reaction times and accuracy are shown both in raw form and corrected for potential confounding factors (trial difficulty, ISI, and feedback valence on the previous trial) are accounted for (the black line in Fig. 7*a* shows residual RT and accuracy after the above confounds have been modeled out using linear and logistic regression, respectively).

**Figure 7.**
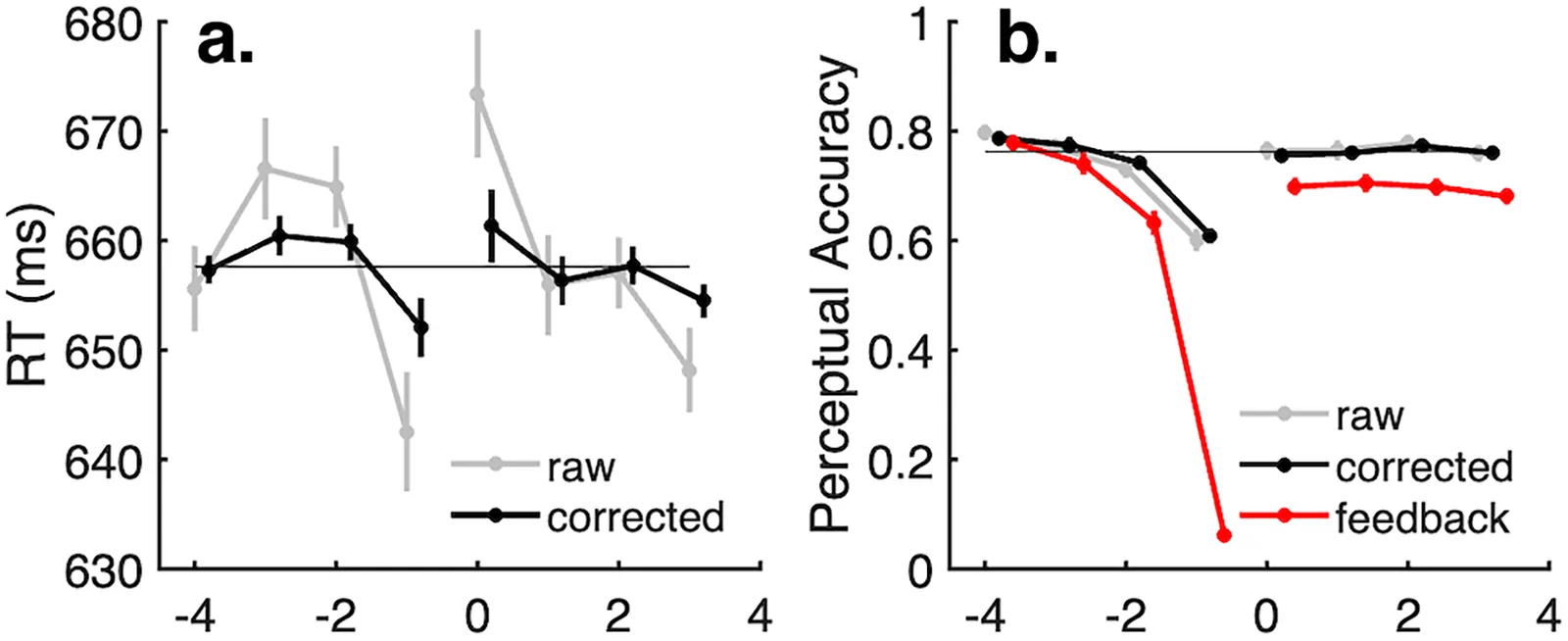
Before correct switches, reaction time decreases but accuracy is maintained. ***a***, Mean reaction time in the four trials before and after a switch; error bars indicate SEM across participants. Gray line, raw mean reaction times. Black line, mean residual reaction times after the effect of task difficulty (angle of rotation); ISI and feedback valence on the previous trial have been removed with linear regression. ***b***, As in ***a***, but here the measure is proportion of trials perceptually correct rather than reaction time. The red line is the proportion of positive feedback—note that switching overwhelmingly follows negative feedback.

Reaction time was significantly reduced on the trial preceding the switch, compared with the grand average over all trials (*t* = −2.05; *p* = 0.050 for corrected reaction times); there was no significant effect on any other trial. There were no significant changes in accuracy.

Note that this analysis was limited to “correct” switches, that is, cases on which participants were working on the irrelevant side (perhaps following a reversal in the ground-truth relevant side) and switched to the relevant side. In the converse case (“incorrect switches”) accuracy was necessarily low on the trial preceding the switch, presumably because in this case only inaccurate trials would generate negative feedback, which in turn triggered switching.

#### Physiological measures

Having established that the decision to switch was both affected by properties of the current stimulus and evolved over trials due to reward history, we then sought to link these two phenomena to beta-band activity in the EEG and pupil dilation.

### Beta oscillations

We defined two time windows for statistical analysis of beta-band activity—a premovement window, from 1,000 to 500 ms before movement onset, and a postmovement phase, from 500 to 1,000 ms after movement onset.

Throughout the analysis of EEG data, we used the log of beta power as the dependent variable as log(beta) was approximately normally distributed.

### Pupil dilation

We log transformed our measure of pupil size (pupil area, expressed as a percentage of the session average pupil area). We also downsampled the pupil data, which were recorded at 1,000 Hz, by averaging in 100 ms bins, to increase SNR.

#### Pre- but not postmovement beta and phasic but not tonic pupil dilation reflect motor readiness

The phasic motif of premovement beta dip and postmovement beta rebound has been posited to control action release and termination. Quantitatively, the amplitude of the premovement beta power reduction predicts motor readiness (reaction time and ability to cancel actions), but such evidence is lacking for the postmovement beta rebound. Therefore we first investigated whether response readiness predicted beta-band power both pre- and postmovement.

### Predicted and residual RT

Before relating reaction time to beta power and pupil dilation, we first partitioned reaction time into a component that could be predicted based on task variables (perceptual difficulty and the duration of the working memory interval; see Eq. 2 in Materials and Methods) and the (substantial) residual variation that could not be predicted using task variables; we therefore defined “residual RT” as the difference between the actual reaction time and that predicted based on perceptual difficulty and working memory interval:

$$\mathrm{RT}=\beta_{0}+\beta_{1}\alpha +\beta_{2}\mathrm{ISI}+Residual\;RT.\;(9)$$

(Definition of residual RT)

Residual RT likely reflects endogenous factors such as motor readiness or level of alertness; notably residual RT was higher on trials when participants made perceptual errors (paired *t* test; $t_{\left(26\right)}=\;-7.99;p<0.0001$), perhaps because participants were in a less attentive state on these trials. This effect was highest when errors were made on easy trials (interaction of perceptual correctness and difficulty: $t_{\left(26\right)}=\;-3.78;\;p<0.0001$)—presumably, errors on easy trials occurred only in the most inattentive states.

### Premovement beta depends on predicted and residual RT

Premovement beta power reduction is associated with greater motor readiness [faster reaction times and reduced ability to cancel actions (He et al., 2020; Wessel, 2020)]. Task factors affected reaction time, namely, the angle of rotation (determining difficulty) and the duration of the working memory interval (ISI). We expected the speedier responses associated with easy perceptual decisions and long ISIs to be associated with greater premovement reduction in beta power. However, reaction time varies over and above the expected effects dependent on task variables; we asked whether this residual RT, which putatively reflects endogenous variation in response readiness, also affects the depth of the premovement beta dip.

We performed a linear regression predicting beta power in the premovement period, based on the elements of predicted RT (the angle of rotation and the working memory interval, termed ISI), and the measured residual RT:

$$\log(\beta_{\mathrm{pre}})\;∼\;[\mathrm{constant}+\;\alpha \;+\;\mathrm{ISI}\;+\;\mathrm{residual}\;\mathrm{RT}],\;(10)$$

(Linear regression predicting beta power by response readiness)

where α is the angle of rotation between Stimuli 1 and 2 (equivalent to perceptual difficulty), ISI is the duration of the working memory interval, and residual RT is the difference between actual reaction time and the value predicted based on α and ISI in Equation 9 above.

We observed that factors which increased response readiness (longer working memory interval and lower perceptual difficulty) were reflected in increased beta power reduction in the premovement phase, as was our measure of unpredicted variation in response readiness (residual RT). Statistically, we carried out group-level *t* tests on the coefficients from the participant-level GLMs (as per Eq. 10); time points where *p* < 0.05 with and without Bonferroni’s correction for 81 timepoints are indicated by heavy and light lines in Figure 7, respectively.

### Postmovement beta does not depend on predicted and residual RT

We then asked whether the converse effect was present in the postmovement period. We might have expected greater postmovement beta on trials with greater response readiness, reflecting a movement termination process matched to the vigorous movement initiation. In fact, the factors affecting premovement beta had little effect in the postmovement phase, with only a briefly significant effect of working memory interval evident in this time period (Fig. 7).

### The phasic pupil response decreases when “response readiness” is higher

Mirroring the analysis of beta power, we used a time-resolved GLM to investigate whether pupil dilation varied with the “response-readiness factors” that drove reductions in premovement beta power:

log(pupil)∼[constant+α+ISI+residualRT].(11)

(GLM response readiness—pupil)

Pupil size was increased on trials with high residual RT (4)—this effect was significant (*p* < 0.05 two tailed, Bonferroni-corrected for 100 timepoints) for a window from 550 ms before movement onset to 2,050 ms afterward. This represents a modulation of the phasic component of the pupil response (Aston-Jones and Cohen, 2005).

Neither of the task factors driving “predicted RT” (difficulty and working memory interval or ISI) had a statistically significant effect on pupil size at any time point, suggesting that endogenous variations in arousal and motor readiness, rather than task factors were the key factor associated with pupil size in this phase.

#### State inference is reflected in postmovement beta and tonic beta power

Next we asked whether the detection of state reversals (i.e., the changes in which stimulus gave deterministic feedback that participants had to track) was reflected in changes in beta power or pupil dilation.

Previous research (Tan et al., 2016) suggests that the postmovement beta rebound reflects a mechanism by which feedback is permitted or blocked from updating a motor model. However, in the current task, model updating or state inference involved integrating over multiple trials (because the meaning of feedback was modified by perceptual uncertainty) and the model to be updated was more abstract.

Behavioral analysis (lagged regression, described above) confirmed that participants accumulate evidence of a state reversal over at least two trials**.** We therefore reasoned that a component associated with state inference might be sustained over multiple trials in the present task.

To test this hypothesis, we ran a time-resolved GLM in which feedback valence on the current and previous trials were included as an explanatory variable (in addition to the response-readiness factors included in Eq. 10):

$$\log\;(\beta_{t})∼\;[\mathrm{constant}+\;x_{t}+\;x_{t-1\;}+\alpha \;+\mathrm{ISI}\;+\mathrm{residual}\;\mathrm{RT}],\;(12)$$

(Linear regression predicting beta power based on feedback history)

where, as in previous equations, α is the angle of rotation between Stimuli 1 and 2 (equivalent to perceptual difficulty), ISI is the duration of the working memory interval, and residual RT is the difference between actual reaction time and the value predicted based on α and ISI as defined in Equation 9. $x_{t}$ denotes feedback on trial *t*, coded as 1 if feedback is positive and 0 if feedback is negative.

We found that indeed, there was a postfeedback decrease in beta power when the feedback was negative.

Additionally, there was a sustained decrease in beta power throughout the trial when the previous trial has received negative feedback (making the observer less confident/more willing to update) rather than positive feedback (making the observer more confident in their model; Fig. 3*a*,*b*). We refer to this sustained decrease in beta power, which builds up over several trials, as a “tonic beta” effect.

### Postmovement beta power decreases around the time of a state reversal

We next asked whether the feedback-related effects built up systematically around state reversals—state reversal was defined from participant behavior, as the point at which participants switch from responding on one side, to the other. Visualization of beta power on the trials preceding and following a switch (Fig. 3*c*,*d*) shows a large decrease in postfeedback beta on the final trial before the side switch. Postfeedback beta power remains low after the side switch and returns to baseline gradually over several trials. The reduction in beta power following the side switch is sustained across the whole trial (Fig. 3*e*,*f*—summarized in Fig. 3*d* as the average beta power in the time window 1–0.5 s before the movement on each trial). Trials on which beta power in each time window differed significantly from the whole-task average (for that time window) were identified using a general linear model in which the effects of feedback valance, previous-trial feedback valance, task difficulty, and residual RT were included as confound regressors (the red and black traces in Fig. 3*c* reflect beta power corrected for these confounds). Results were Bonferroni-corrected for eight comparisons (four trials before and four trials after the state switch).

In summary, after a negative-feedback event, there was an immediate reduction in beta power (seen in the postmovement phase in Fig. 3*a*). The reduction in beta power was sustained as a tonic effect throughout the following trial (seen in Fig. 3*b*). In the run-up to a side switch, each negative feedback resulted in a further reduction in tonic beta power (Fig. 3*c*, red trace), which was sustained and compounded over subsequent trials (Fig. 3*c*, black and red traces). Following a side switch tonic beta power gradually returned to a higher level, apparently “stepping up” each time a positive feedback event occurred (Figs. 3*c*).

### Tonic beta power tracks the evidence for a state reversal

To provide a more mechanistic characterization of the changes in beta power, we constructed an optimal Bayesian observer model (see Materials and Methods) that inferred, given the feedback and sensory uncertainty on each trial, the probability that the currently selected side was the deterministic side. The prior and posterior belief that the currently selected side is the correct (deterministic) one is plotted in Figure 3*d*. This belief falls gradually over a number of trials but falls dramatically between the prior and posterior on the final trial before a (behavioral) reversal. The strength of belief that the currently selected side is indeed correct (“state belief”) therefore strongly resembles the time course of the tonic beta effect.

### No significant effects of task variables on lateralized beta power

While beta power is often studied as a lateralized phenomenon, in the current task, by design, the response hand could not be predicted on a given trial in advance of the actual response. Therefore, we did not expect to see lateralized effects of state inference. For completeness, we tested for effects of predicted RT, residual RT, and trials until switch, using lateralized beta power as the response variable (left–right reversing topographies for right-hand response trials to allow for averaging across all trials). As expected, on average across all trials, we observed a lateralized version of the perimovement beta motif (premovement decrease in beta power followed by a postmovement beta rebound, contralateral to the response hand), which was located anterior to the observed nonlateralized effects, being most prominent over central electrodes (C1/2, C3/4, CP1/2, and CP3/4). Lateralized beta power around the time of the button press was not affected by state inference (contra-ipsi beta power did not depend on the number of trials until a switch). There was a trend toward an effect of motor readiness (residual RT) on lateralized beta power, in the rising phase between the perimovement minimum and postmovement rebound; this effect lay outside of preplanned time windows and was not significant with multiple-comparisons correction for all timepoints.

#### No posterror slowing effects in behavior or beta power

Following a negative-feedback trial, we might expect decreased response readiness (posterror slowing) and a concomitant increase in premovement beta power. In fact, there was no significant increase in reaction time following negative feedback on either perceptually correct trials (*t*_(26)_ = −0.95; *p* = 0.35, two tailed, when negative feedback on the previous trial was added as a coregressor to Eq. 10) or perceptually incorrect trials (*t*_(26)_ = 1.00; *p* = 0.33, two tailed). If there had been a behavioral posterror slowing effect, we would have expected to see an increase in premovement beta power (reflecting increased response inhibition); since there was no behavioral posterror slowing effect, this was not necessarily expected. However, premovement beta power actually showed the opposite effect, that is, it decreased following negative feedback (effect of previous-trial feedback, $x_{t-1}$ in the model updating GLM, Eq. 12; Fig. 3*b*). Rather than a change in response caution, this sustained decrease in beta power following negative feedback may reflect that the state model is susceptible to updating.

*Tonic pupil changes also track state uncertainty, mirroring changes in tonic beta*. The effects seen for tonic beta power are reminiscent of pupil dilation effects in which the baseline (tonic) pupil size increases around the time of state reversals (Nassar et al., 2012; Browning et al., 2015; Muller et al., 2019).

Therefore, mirroring the analyses performed on beta power, we asked whether feedback on the current and previous trials affected pupil size:

$$\log\;(\mathrm{pupi}l_{t})∼\;[\mathrm{constant}+x_{t}+x_{t-1}+\alpha \;+\mathrm{ISI}\;+\mathrm{residual}\;\mathrm{RT}].\;(13)$$

(Linear regression predicting pupil size based on feedback history)

The results mirrored the effects seen for tonic beta power. After a negative-feedback event, there was an immediate increase in tonic pupil size (seen in the postfeedback phase in Fig. 4*a*). The increase in pupil size was sustained as a tonic effect throughout the following trial (seen in Fig. 4*b*). In the run-up to a side switch, each negative-feedback event resulted in a further increase in tonic pupil size (Fig. 4*c*, red trace), which was sustained and compounded over subsequent trials (Fig. 4*c*, black and red traces). Following a side switch, tonic pupil size gradually returned to a lower level, apparently “stepping down” each time a positive feedback event occurred (Fig. 4*c*). As for tonic beta power (above), the changes in tonic pupil size reflect closely the strength of belief that the alternative side is indeed correct (“state belief”) from an optimal Bayesian model (Fig. 4*d*). Note that here we plot the strength of belief against the currently selected side (which is exactly the complement of the parameter plotted for beta power in Fig. 3) to match the polarity of the pupil dilation effect and therefore aid visual comparison.

*Postfeedback beta power mediates the effects of task on pupil dilation*. The parallel effects observed for beta power and pupil dilation hint at a common neural mechanism driving both EEG and pupil effects. To investigate this hypothesis, we conducted two formal mediation analyses.

Firstly, we asked whether the effect of feedback valence on pupil dilation in the postfeedback period (2–4 s postfeedback) was mediated by beta power in the postfeedback window (500–1,000 ms after feedback)—this links the parallel EEG and pupillometry results shown in Figures 3, *a* and *b*, and 4, *a* and *b*. Secondly, we asked whether the effect of response readiness (residual RT) on the phasic pupil response (0–2 s after the button press) was mediated by premovement beta power reduction (in the window 1,000–500 ms prior to the button press)—this links the parallel EEG and pupillometry results shown in Figure 2.

### Feedback effects

Following the procedure of Baron and Kenny (1986), we first confirmed the effect of our independent variable, feedback valence, in the context of all other task factors, on the dependent variable which was pupil size in the postfeedback window (2–4 s postfeedback, shaded region on Fig. 4*a*,*b*,*d*,*f*): this is the analysis in Equation 13 and the effect of the independent on dependent variable was significant in the chosen time window—the pupil is larger after negative feedback ( $t_{\left(26\right)}=6.69;p<0.005$).

We then verified that the independent variable, in the context of the other task variables, predicts the putative mediator, postfeedback beta power (in the window 500–1,000 ms postfeedback). This is the analysis in Equation 11, and the effect of the independent on mediator variable was significant—the beta power is lower after negative feedback ( $t_{\left(26\right)}=7.41;\;p<0.005$).

We then modeled the effect of feedback valence on the pupil size in the context of the mediator (beta power):

$$\begin{array}{rl} \log\;(\mathrm{pupi}l_{t})∼[ & \mathrm{constant}+\;x_{t}+\;x_{t-1\;}+\;\alpha \;+\;\mathrm{ISI}\;\\ & +\;\mathrm{residual}\;\mathrm{RT}+\;log(\beta_{t})]. \end{array}\;(14)$$

(Mediation of feedback effect on pupil by beta power)

Using a paired *t* test, we asked whether the coefficient for the effect of feedback valence on pupil size was significantly reduced in the presence of the mediator. This was indeed the case $\left(t_{\left(26\right)}=2.96;\;p=\;0.0067\right)$, indicating that beta power significantly mediates the effect of feedback valence on pupil dilation.

#### Response-readiness effects

Next, we asked whether the effect of response readiness (residual RT) on the phasic pupil response (0–2 s after the button press) was mediated by pre movement beta power reduction (in the window 1,000–500 ms prior to the button press). Following the Baron–Kenny procedure as above, we verified that the effect of residual RT on pupil size was significant (Eq. 11)—it was $t_{\left(26\right)}=6.53;p<0.005$. We also verified that residual RT predicted beta power in the premovement interval (Eq. 10)—it was $t_{\left(26\right)}=6.33;p<0.005$. We then asked whether there was a significant mediation effect, using a paired *t* test to compare the coefficients for the effect of residual RT on pupil size with and without the inclusion of premovement beta power:

$$\log\;(\mathrm{pupi}l_{t})∼[\mathrm{constant}+\;\alpha \;+\mathrm{ISI}\;+\mathrm{residual}\;\mathrm{RT}+\;\log\;(\beta_{t})].\;(15)$$

(Mediation of response-readiness effect on pupil by beta power)

However there was no reduction ( $t_{\left(26\right)}=-0.42;\;p=\;0.68$) indicating that the effects of response readiness (residual RT) on the prestimulus beta power and the phasic pupil response are independent.

#### Time-resolved relationship between beta power and pupil dilation

Finally, to visualize the relationship between beta power and pupil size, we used a two-dimensional time-resolved regression in which we predicted pupil size at each timepoint i from beta power at each timepoint j:

$$\log\;(\mathrm{pupi}l_{i})∼[\mathrm{constant}+\log(\beta_{j})].\;(16)$$

(Predicting pupil size from raw beta power)

It can be seen (Fig. 8*a*) that increased beta power in the postfeedback period (in the time period in which updating effects were observed) was associated with increased pupil dilation throughout the postfeedback epoch, perhaps reflecting a state in which model updating is enabled.

**Figure 8.**
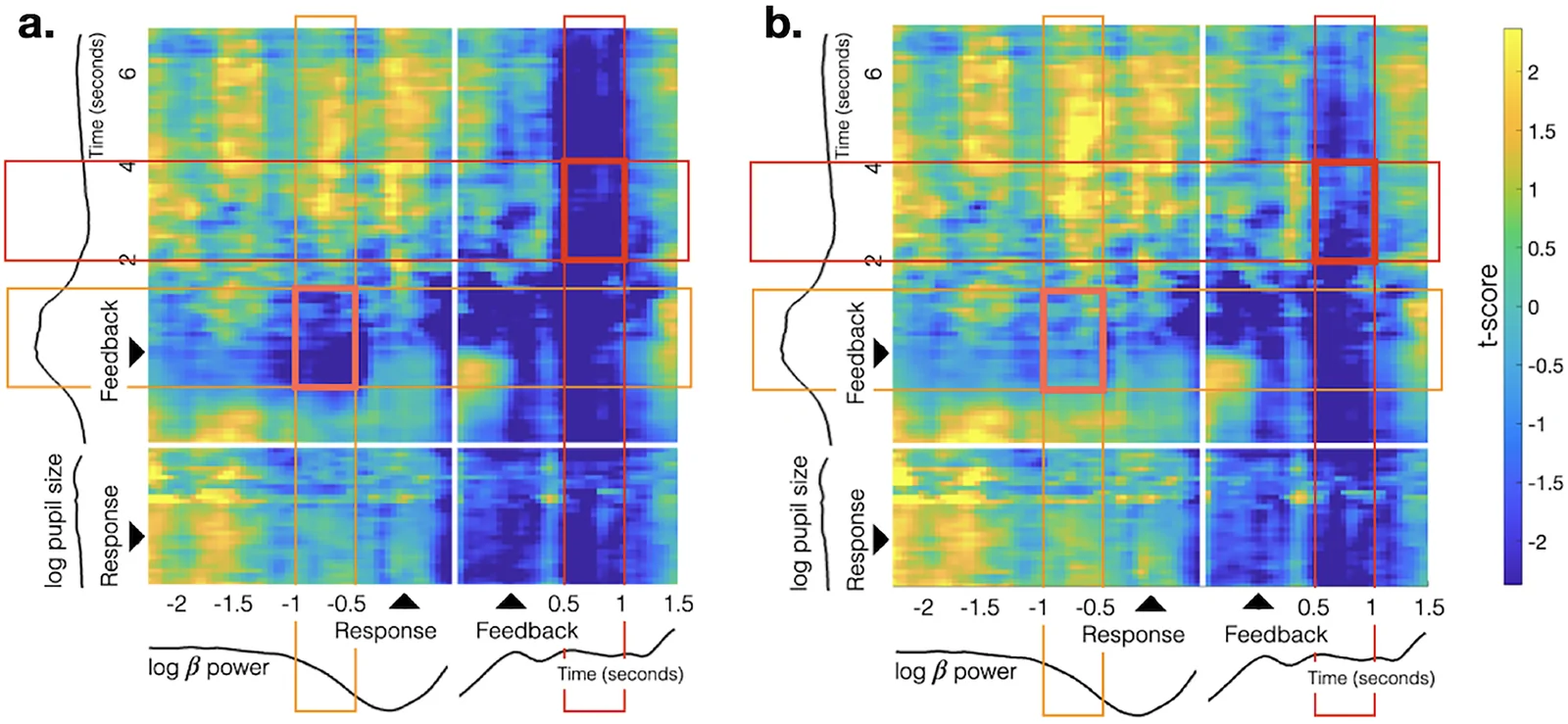
*t* scores from a 2D time-resolved regression in which beta power is used to predict pupil size. The marginal traces are the mean beta power and pupil size over all trials (similar to the traces in Figs. 3, 4), shown for context. Saturated colors are significant at *p* < 0.05 two tailed, correcting for two multiple comparisons (with the plan to look at the two predefined pairs of time windows, as defined in the mediation analysis: EEG 1,000–500 ms preresponse against pupil 0–2,000 ms postresponse (response-readiness analysis—orange boxes) and EEG 500–1,000 ms postfeedback against pupil 2,000–4,000 ms postfeedback (feedback valence analysis—red boxes). ***a***, This figure, shown for illustration of the full time-resolved relationship between pupil and beta power, indicates that the response-readiness effects are phasic in nature—premovement beta power is related only to the phasic pupil response (note lack of saturated colors elsewhere in the vertical orange box). In contrast, feedback-related effects are sustained over time—postfeedback beta power has a relationship with pupil size throughout the trial, reflecting the tonic nature of this effect. ***b***, Analysis as in panel ***a*** but now showing the residual relationship between beta power and pupil size when all shared task effects (as in Eqs. 12, 13) are first modeled out of the pupil and EEG data; in other words, the residuals from Equations 12 and 13 are used as the pupil and EEG data in this figure. Note that, in contrast to panel ***a***, once shared task effects are removed, the response-readiness effects (orange boxes) disappear, indicating that these are driven by shared task factors. In contrast, the feedback-related effects (red box) remain, indicating a link between tonic beta power and tonic pupil size that is not explained simply by shared task influences.

Additionally, decreased beta power in the premovement phase (the same time window in which beta power tracks residual RT) was associated with decreased pupil size across most of the trial epoch, perhaps reflecting an overall “on task” or focused state (Aston-Jones and Cohen, 2005).

## Discussion

We examined distinct computational influences on beta power: motor readiness, sensory feedback and model updating, and their parallels in pupil dilation.

The results suggest that beta oscillations and pupil dilation operate on two distinct timescales, with different computational drivers and consequences. Firstly (as expected), a “phasic” effect is seen: in the few hundred milliseconds around movement release, there is a transient suppression of the beta rhythm (before the movements) and rebound (after the movement) which are associated with movement release and termination. This effect is paralleled by changes in the size of the phasic pupil response. Secondly, slower “tonic” variations in beta power and pupil size occur spanning multiple movements over tens of seconds. These tonic changes appear to track the strength of evidence for a state reversal (as captured in a Bayesian ideal observer model). The tonic beta and pupil effects seem to reflect a common underlying neural mechanism, as the effects of task on pupil size are statistically mediated by tonic beta power. This tonic effect is much less well known, to date, than links between phasic beta and response readiness.

It is established in the literature and reconfirmed here that motor readiness and arousal primarily affect the phasic beta time course; specifically, premovement beta power reduction is strongly associated with motor readiness. When the premovement beta dip is large, reaction times are faster, and participants are less able to “cancel” planned actions [as in the stop signal task (Swann et al., 2009; Wessel, 2020)]. Movements initiated during the beta rebound caused by a previous movement (i.e., when beta activity is high) are slower (Muralidharan and Aron, 2021). Brain stimulation studies confirm the causal role of premovement beta desynchronization in movement speed: Volitional decrease of movement-related beta via neurofeedback produces faster movements (He et al., 2020), and transcranial entrainment of beta oscillations via beta-frequency alternating current stimulation (TACS) slows movement (Pogosyan et al., 2009). In the present study, as expected, premovement beta power correlated with motor readiness, operationalized as residual variation in reaction time that could not be explained by task factors.

Interestingly, motor readiness also correlated with the size of the phasic pupil response, perhaps reflecting a relationship between pupil size and arousal (Aston-Jones and Cohen, 2005). However, statistical mediation analysis suggested that phasic beta and phasic pupil correlates of response readiness may have independent mechanisms, as there was no evidence that beta mediates the link between response readiness (as measured by reaction time) and pupil size.

The key finding of this study is that a second component of the beta rhythm reflects a more abstract or higher-order feature of the environment—beta power declines tonically over the course of several trials as participants accumulate that their (abstract) model of the environment should be updated and rises gradually over several trials after such an update. This is mirrored by changes in tonic pupil dilation, and these two biomarkers may reflect a common underlying mechanism, as formal mediation analysis shows that changes in tonic beta mediate the effect of task on tonic pupil size. The changes in tonic beta power and pupil dilation closely resemble the strength of evidence for a state reversal as captured using a Bayesian ideal observer model.

We describe the model-update effects on beta power and pupil dilation as tonic, because they are long lasting, spanning multiple trials, and is sustained independently of the time course of individual actions or trials; this is distinct from the phasic premovement beta desynchronization and postmovement beta rebound which form a characteristic movement-locked time course and the phasic pupil effect, a characteristic sharp dilation and contraction of the pupil that occurs after movement release. However, the model-update component in tonic beta does appear to have a close link with the phasic beta rebound: When feedback is received that conflicts with the current model of the environment, the beta rebound is suppressed. This suppression is carried over into a tonic effect.

The results are concordant with previous findings that phasic postmovement beta power is linked to model updating. It has previously been reported that the postmovement beta rebound is decreased when feedback should be permitted to update an internal model and increased when feedback should be blocked. In a sensorimotor context, the beta rebound is reduced (1) when there is a prediction error (Tan et al., 2014), (2) when the internal model is uncertain because of noise (Tan et al., 2016), and (3) when the sensory feedback is relatively certain (low noise; Palmer et al., 2019). Beyond the sensorimotor framework, beta power in the occipital cortex is reduced when visual feedback should be used to update a model and increased when visual feedback should be ignored (Limanowski et al., 2020). In decision-making, the beta rebound is reduced when exploratory (rather than exploitative) choices are made and especially when the feedback confirms that switching preference to the explored option would be beneficial (Chernyshev et al., 2023).

The tonic beta effect may be more evident in the present study than in previous work because of differences in the task design. Most previous reports of update-related modulations of the beta rhythm come from visuomotor adaptation paradigms, in which model update tends to be strongly correlated with feedback valence (and magnitude), and the model is continuously updated. In contrast, we used a hierarchical perceptual decision-making task designed to partially dissociate model updating from both feedback valence and error magnitude. The two key features of the task were that negative feedback should be given a variable weight depending on the difficulty of the perceptual decision and that the detection of state switches (need for model update) depended on the integration of several feedback events, culminating in rare “complete switches” of the model rather than incremental updating.

Two studies have reported effects in the premovement “beta desynchronization” period, consistent with our observed tonic effect. (1) In a sensorimotor adaptation study, Palmer et al. (2019) showed that the premovement beta desynchronization was increased when model uncertainty was high (following a sensorimotor perturbation). (2) In a decision-making study, Chernyshev et al. (2023) found that (phasic) predecision beta power was reduced when people made exploratory decisions. In both cases, because trial-wise baseline correction (or mean correction) was employed, tonic effects as reported in the present manuscript would be less apparent and might appear only as modulations of phasic effects.

In the premovement beta phase, we observe a juxtaposition of two apparently independent effects on beta power. The magnitude of the phasic premovement beta dip was most closely linked to trial-by-trial variations in motor readiness, independently of model certainty; however, we also recorded effects in the premovement beta period of model updating, as a part of a long-timescale, tonic effect on beta power. This effect was independent of motor readiness effects; indeed, tonic beta power was suppressed following a model update (Fig. 2*c*), but in this same time period, reaction times are not affected (Fig. 7). This juxtaposition suggests two functionally distinct components to the beta rhythm, even within the same (prestimulus) time period—as the tonic beta suppression associated with model updating does not result in faster reaction times, even though beta power is in fact lower in the prestimulus window associated with motor readiness.

There are two possible interpretations of this two-timescale beta phenomenology. One is that a single beta generator tracks both readiness to respond to and update the model underlying our responses; as these processes naturally happen on different timescales, modulation of beta on two timescales is seen. An alternative explanation is that beta power as measured in the scalp EEG is an overlay of contributions from two sources, associated with motor readiness/gating and model updating, respectively. MEG studies using beamforming have previously suggested that premovement beta desynchronization and postmovement beta rebound may be topographically dissociable: Beta rebound tends to originate from a relatively anterior source, and beta desynchronization originates from a relatively posterior region in the sensorimotor cortex (Jurkiewicz et al., 2006; Fry et al., 2016; Pakenham et al., 2020). Outside the field of beta oscillations, model updating is associated with activity in the medial prefrontal cortex and anterior cingulate cortex (Holroyd and Coles, 2002; Alexander and Brown, 2011; O’Reilly et al., 2013) which lie anterior to the motor cortex. The present study, using scalp EEG which has relatively poor ability to dissociate neural sources, cannot distinguish between sources of the tonic and phasic components; however the independent influences of phasic (premovement) beta and tonic beta on reaction time and the differential relationship of tonic and phasic beta power to pupil dilation argue in favor of a two-source model.

## Data Availability

All codes are available on request by emailing the corresponding author.
